# Effectiveness of sepsis bundle application and outcomes predictors to cirrhotic patients with septic shock

**DOI:** 10.1186/s12879-021-06194-5

**Published:** 2021-05-26

**Authors:** Yong-Ye Yang, Yin-Chou Hsu

**Affiliations:** 1 Department of Emergency Medicine, E-Da Hospital, I-Shou University, No.1, Yida Road, Jiao-su Village, Yan-chao District, Kaohsiung City, 82445 Taiwan; 2 School of Medicine for International Student, I-Shou University, Kaohsiung, Taiwan

**Keywords:** Liver cirrhosis, Septic shock, Prognosis, Care bundles, Emergency department

## Abstract

**Introduction:**

Cirrhotic patients with septic shock have a poorer prognosis compared with the general population. Our study aimed to investigate the survival benefit of the implementation of hour-1 bundle proposed by Surviving Sepsis Campaign, and to analyze the predictors associated with short-term mortality of these patients.

**Methods:**

A single-center, retrospective case-control study was conducted among adult patients who visited the emergency department between January 1, 2018 and December 31, 2019. All patients with a diagnosis of liver cirrhosis and septic shock were enrolled. Their baseline characteristics, laboratory results, source of sepsis, and sepsis bundle management were recorded. We further divided the patients into survivor and non-survivor groups to identify independent prognostic factors.

**Results:**

A total of 88 patients were eligible for this study. The overall 30-day mortality rate was 53.4% (47/88). The proportion of hour-1 bundle achievement was 30.7% (27/88). There were no significant mortality differences between the hour-1 bundle achievement and non-achievement groups (44.4% vs. 57.4%, *p* = 0.35). Compared with the patients in the survivor group, patients in the non-survivor group had significantly more advanced stage of cirrhosis and a lower proportion of receiving source control (4.3% vs. 22.0%, *p* = 0.02). The chronic liver failure-sequential organ failure assessment (CLIF-SOFA) score (adjusted hazard ratio [AHR] =1.52, *p* < 0.01), serum lactate (AHR =1.03, *p* < 0.01), and source control (AHR =0.54, *p* = 0.02) were identified as independent prognostic factors in the multivariate regression model. Furthermore, the CLIF-SOFA score (area under curve [AUC]: 0.81) and lactate levels (AUC: 0.77) revealed good mortality discrimination ability in cirrhotic patients with septic shock.

**Conclusions:**

The application of the hour-1 bundle did not reveal a significant survival benefit to cirrhotic patients with septic shock. Clinicians could utilize CLIF-SOFA scores and lactate levels for mortality risk stratification and put more emphasis on the feasibility of source control to improve their prognosis.

## Introduction

Cirrhosis is the pathological formation of regenerative nodules surrounded by fibrous bands in response to chronic liver injury. This results to portal hypertension and hepatic synthetic dysfunction, which is the 14th most common cause of death in adults worldwide [1, 2]. Bacterial overgrowth, increased intestinal permeability, and cirrhosis-associated immune dysfunction predispose cirrhotic patients to bacterial infection, leading to a four-fold increased mortality compared with non-cirrhotic patients [2, 3]. Bacterial infection is also an important precipitant of acute-on-chronic liver failure (ACLF), which is characterized by acute liver dysfunction and one or more extrahepatic organ failure, resulting to increased short-term mortality [4].

Sepsis and septic shock is a life-threatening organ dysfunction caused by dysregulated host responses and is a leading cause of critical illness and mortality worldwide [5]. According to the updated practice guidelines proposed by the *Surviving Sepsis Campaign (SSC)*, a care bundle of sepsis treatment that includes serum lactate measurement, blood culture collection, broad-spectrum antimicrobial agent administration, fluid resuscitation, and vasopressor support should be achieved within an hour after sepsis recognition [6]. Cirrhosis was identified as a precipitant of sepsis and an independent poor prognostic factor in patients with sepsis and septic shock [7]. Importantly, whether cirrhotic patients with sepsis and septic shock could benefit from the sepsis bundle implementation has rarely been validated [8]. There was only one study addressing the effectiveness of 6-h sepsis bundle application in cirrhotic patients with septic shock, which did not provide survival improvement [8]. Compared with other subgroup analyses in patients with sepsis, the paucity of studies focused on patients with cirrhosis and sepsis, particularly in those with septic shock [9–12]. The goal of this study was to describe the characteristics and investigate the survival benefit of the SSC hour-1 bundle, and to analyze the independent factors associated with short-term mortality in cirrhotic patients with septic shock.

## Methods

### Study design

This retrospective observational case-control study was conducted at a tertiary referral medical center located in southern Taiwan with approximately 58,000 emergency department (ED) visits per year. The study was approved by the local institutional review board (EMRP-108-144), and all anonymized clinical information was collected from electronic medical record systems between January 1, 2018 and December 31, 2019. All adult patients (aged ≥18 years) with septic shock and liver cirrhosis who visited the ED during the studied period were enrolled. The diagnosis of septic shock was identified in accordance with Sepsis-3 definitions: sepsis with persistent hypotension requiring vasopressors to maintain a mean arterial pressure (MAP) of ≥65 mmHg, and a serum lactate level > 2 mmol/L [5]. Patients with cirrhosis were recognized by the ED diagnostic code (ICD-10) and diagnosis was further confirmed with results of liver biopsy or a combination of clinical and image examinations (e.g., presence of ascites, encephalopathy). The treatment of septic shock was initiated in accordance with the hour-1 bundle proposed by SSC: 1) measure serum lactate, 2) obtain blood cultures prior to administration of antimicrobial agents, 3) administer broad-spectrum antimicrobial agents, 4) administer 30 ml/kg crystalloid rapidly for hypotension, 5) apply vasopressors if patients are hypotensive during or after fluid resuscitation to maintain MAP ≥65 mmHg [6]. Patients with prior liver transplantation, inter-facility transfer, or those who had a cardiac arrest event before ED arrival were excluded. This study protocol followed the STROBE guidelines [13], and as the retrospective observational nature of the study, the ethics committee waived the requirement for informed consent.

### Data collection

All eligible patients were divided into survivor (alive to discharge) and non-survivor (mortality during hospitalization) groups for further analysis. Their laboratory results were obtained within 6 h of septic shock recognition. Model for End-stage Liver Disease (MELD) score, Child-Pugh score, chronic liver failure-sequential organ failure assessment (CLIF-SOFA) score, and presence of ACLF were calculated based on the laboratory results [14, 15]. The source of sepsis was classified as: respiratory tract infection (increased infiltration revealed on chest radiograph combined with clinically compatible symptoms), urinary tract infection (urinalysis revealed pyuria and bacteriuria), spontaneous bacterial peritonitis (diagnostic paracentesis with leukocyte count of ≥250 cells/μL), biliary tract infection, soft tissue infection, and others according to the corresponding discharge diagnosis of each patient. The source control (e.g., debridement for soft tissue infection, drainage of intra-abdominal abscess, endoscopic removal of obstructed biliary tract) was recorded according to the medical records. The timing of vasopressor and first dose of antimicrobial agent administration was calculated based on the time interval between the recognition of septic shock and drug administration. The amount of fluid administration was calculated during the time interval between the septic shock recognition and initiation of vasopressor support.

### Definitions

The Systemic Inflammatory Response Syndrome (SIRS) criteria were defined according to the 2012 SSC [16]. The quick Sepsis Related Organ Failure Assessment (qSOFA) score was calculated using the initial ED triage variable: Glasgow coma score < 15, respiratory rate ≥ 22 breaths/min, and systolic blood pressure ≤ 100 mmHg [5]. A qSOFA score of ≥2 points was used as the prognostic cutoff value based on previous studies [17]. For culture-positive septic shock, the initial antimicrobial agent was considered appropriate if the cultured pathogen (e.g., blood, urine, sputum) was susceptible to it based on the results of an in vitro susceptibility test. For culture-negative septic shock, the initial antimicrobial agent was considered appropriate if the agent was consistent with generally accepted norms and principles according to the practice guideline [18].

### Outcome measurement and statistical analysis

The primary outcome of this study was to investigate the survival benefit of the hour-1 bundle application in the cirrhotic patients with septic shock. The secondary outcome was to identify independent factors associated with in-hospital mortality in these patients. The significance of difference between continuous variables was determined using the two-sample t-test or Mann-Whitney test for those with and without normal distribution, whereas that between categorical variables was assessed using the chi-square test or Fisher’s exact test. To identify the independent variables associated with short-term in-hospital mortality in patients with cirrhosis and septic shock, we employed a Cox proportional hazards regression model to calculate unadjusted and adjusted hazard ratio and 95% confidence interval. The univariates with a *p*-value < 0.1 were variables included into the regression model. Age and sex were included in the model irrespective of the *p*-value in the univariate analysis. The proportional hazard assumption was tested on the basis of Schoenfeld residuals after fitting the Cox regression model. We also tested the mortality discriminative ability of those independent factors by using area under a receiver operating characteristic (ROC) curves. All data were analyzed using the Statistical Package for the Social Sciences version 22.0 and MedCalc version 18.2.1 software. All statistical testing was two-tailed with significance set at the *p*-value < 0.05.

## Results

**Fig. 1:**
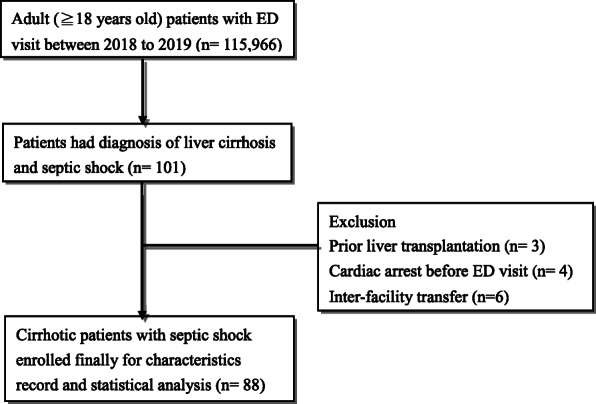
Flowchart of patient enrollment

As shown in Fig. 1, a total of 115,966 adult patients visited the ED during the study period, wherein 101 patients met the selection criteria. After excluding patients with prior liver transplantation (*N* = 3), inter-facility transfer (*N* = 6), and cardiac arrest event before ED arrival (*N* = 4), the remaining 88 patients with cirrhosis and septic shock were finally analyzed.

### Demographic and clinical characteristics

The overall 30-day in-hospital mortality rate of our patients was 53.4% (47/88). As shown in Table 1, the mean age of the patients was 56.3 ± 11.1 years, and most (80.7%) were male. More than half (53.4%) of the patients had cirrhosis attributed to alcohol causes, and the majority of them belonged to advanced cirrhosis stage, according to the Child-Pugh and MELD scores. Most patients (75.0%) met the SIRS criteria, whereas a small portion (30.7%) belonged to the high qSOFA group. Half of the patients (51.1%) developed ACLF, which was in line with their high mean CLIF-SOFA score (11 ± 3).

**Table 1:** Baseline characteristics of cirrhotic patients with septic shock (*n* = 88)

Characteristics	All (*n* = 88)	Non-survivors (*n* = 47)	Survivors (*n* = 41)	*p* value
Age, y, mean ± SD	56.3 ± 11.1	57.0 ± 12.1	55.4 ± 10.0	0.52
Male, n (%)	71 (80.7)	38 (80.9)	33 (80.5)	1.00
Etiology of cirrhosis, n (%)				
Alcohol	47 (53.4)	27 (57.4)	20 (48.8)	0.52
Viral Hepatitis	25 (28.4)	13 (27.7)	12 (29.3)	1.00
Other	16 (18.1)	7 (14.9)	9 (22.0)	0.42
Child-Pugh Score, mean ± SD	10 ± 2	11 ± 2	9 ± 2	< 0.001^*^
MELD score, mean ± SD	27 ± 7	30 ± 5	24 ± 7	< 0.001^*^
SIRS, n (%)	66 (75.0)	37 (78.7)	29 (70.7)	0.46
qSOFA score ≥ 2, n (%)	27 (30.7)	18 (38.3)	9 (22.0)	0.11
ACLF, n (%)	45 (51.1)	32 (68.1)	13 (31.7)	< 0.01^*^
CLIF-SOFA score, mean ± SD	11 ± 3	13 ± 3	9 ± 3	< 0.001^*^
Comorbidities, n (%)				
Diabetes Mellitus	26 (29.5)	12 (25.5)	14 (34.1)	0.48
Hypertension	20 (22.7)	12 (25.5)	8 (19.5)	0.61
Malignancy	24 (27.3)	13 (27.7)	11 (26.8)	1.00
Cerebrovascular accident	11 (12.5)	5 (10.6)	6 (14.6)	0.75
Obstructive lung disease	20 (22.7)	9 (19.1)	11 (26.8)	0.45
Laboratory results				
Hemoglobin, g/dL, mean ± SD	10.3 ± 2.5	10.4 ± 2.4	10.1 ± 2.6	0.47
Leukocyte, × 10^9^/L, median (IQR)	9.7 (4.7–15.5)	12.5 (5.8–16.1)	7.4 (3.7–13.4)	0.06
Platelet, ×10^9^/L, median (IQR)	72.5 (45.3–121.3)	68 (45.0–116.0)	76 (44.5–130.5)	0.47
INR, median (IQR)	1.6 (1.3–2.0)	1.7 (1.5–2.1)	1.4 (1.2–1.8)	< 0.01^*^
Bilirubin, mg/dL, median (IQR)	5.2 (2.4–12.0)	6.3 (3.2–15.4)	3.7 (2.0–7.9)	0.03^*^
Sodium, mmol/L, mean ± SD	131 ± 8	131 ± 9	131 ± 6	0.86
Lactate, mmol/L, median (IQR)	4.0 (2.3–8.8)	6.3 (3.4–11.2)	2.7 (2.1–4.0)	< 0.01^*^
Creatinine, mg/dL, median (IQR)	2.1 (1.4–2.8)	2.2 (1.5–3.3)	1.6 (1.2–2.3).	0.40

*MELD* Model for End-stage Liver Disease, *SD* standard deviation, *IQR* interquartile range, *SIRS* Systemic Inflammatory Response Syndrome, *qSOFA* quick Sepsis Related Organ Failure Assessment, *ACLF* Acute-on-chronic liver failure, *CLIF-SOFA* Chronic liver failure-sequential organ failure assessment, *INR* International Normalized Ratio

*P < 0.05

### Sepsis source and bundle management

**Fig. 2:**
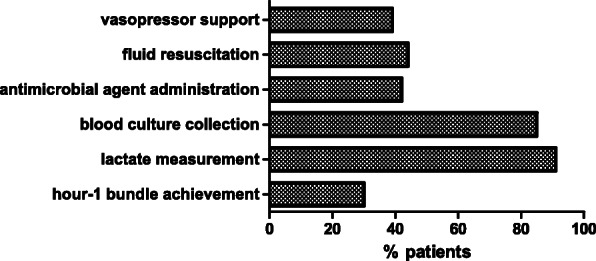
Percentage of patients with compliance of each component of sepsis hour-1 bundle

As shown in Table 2, more than half (59.1%) of the patients had bacteremia (i.e. bloodstream infection). Nearly three-fourths (73.9%) of the patients received appropriate antimicrobial agent administration. Regarding the source of sepsis, spontaneous bacterial peritonitis (34.1%) accounted for the leading cause of infection, followed by respiratory (18.2%) and urinary tract infection (14.8%). As for the sepsis bundle management, the overall proportion of source control implementation was 12.5% (11/88), and the median administration times of first-dose antimicrobial agent and vasopressor were 85 min and 103 min, respectively. In addition, the median amount of crystalloid resuscitation was 1.2 L in our patients. There were 80 (90.9%) and 75 (85.2%) patients received serum lactate measurement and blood culture collection within 1 h of septic shock recognition, respectively; but less than half of the patients received first-dose antimicrobial agent or vasopressor administration within this time limit (Fig. 2). There were also only 39 (44.3%) patients received 30 ml/kg crystalloid infusion for their hypotension. Overall, the proportion of hour-1 bundle achievement was 30.7% (27/88).

**Table 2:** Sepsis source and bundle management of cirrhotic patients with septic shock (*n* = 88)

Variables	All (*n* = 88)	Non-survivors (*n* = 47)	Survivors (*n* = 41)	*p* value
Bacteremia, n (%)	52 (59.1)	27 (57.4)	25 (61.0)	0.83
Infection Source, n (%)				
Respiratory tract infection	16 (18.2)	13 (27.7)	3 (7.3)	0.03^*^
Urinary tract infection	13 (14.8)	5 (10.6)	8 (19.5)	0.37
Spontaneous bacterial peritonitis	30 (34.1)	19 (40.4)	11 (26.8)	0.26
Biliary tract infection	9 (10.2)	1 (2.1)	8 (19.5)	0.01^*^
Soft tissue infection	12 (13.6)	4 (8.5)	8 (19.5)	0.21
Other	8 (9.1)	5 (10.6)	3 (7.3)	0.72
Appropriate antibiotics, n (%)	65 (73.9)	35 (74.5)	30 (73.2)	1.00
Source control, n (%)	11 (12.5)	2 (4.3)	9 (22.0)	0.02^*^
1st dose antibiotics administration time, minutes, median (IQR)	85.0 (44.8–149.5)	94.0 (47.0–150)	70.0 (29.5–165)	0.31
Vasopressor initiation time, minutes, median (IQR)	103.0 (68–151.3)	115.0 (70–175)	80.0 (51–133.5)	0.37
Fluid amount, L, median (IQR)	1.2 (0.7–1.6)	1.0 (0.5–1.5)	1.3 (1.0–1.6)	0.16
Hour-1 bundle achievement, n (%)	27 (30.7)	12 (25.5)	15 (36.6)	0.35

*P < 0.05

### Subgroup analysis

We further divided patients into non-survivor and survivor groups for comparison. As shown in Table 1, patients in the non-survivor group had significantly higher Child-Pugh, MELD, and CLIF-SOFA scores, and a higher proportion of ACLF development compared with patients in the survivor group. Patients in the non-survivor group also had significantly higher serum bilirubin, lactate, and international normalized ratio (INR) (Table 1). Regarding the source of sepsis, a significantly higher proportion of respiratory tract infection and a lower proportion of biliary tract infection were observed in the non-survivor group (Table 2). Notably, patients in the non-survivor group received a significantly lower proportion of source control (4.3% vs. 22.0%, *p* = 0.02).

### Outcome measurement

The components of the hour-1 bundle included the timing of antimicrobial agent and vasopressor administration, and the amount of fluid resuscitation, of which all revealed non-significant differences between the non-survivors and survivors groups (Table 2). There was also no significant mortality difference between the hour-1 bundle achievement and non-achievement groups (44.4% vs. 57.4%, *p* = 0.35).

**Fig. 3:**
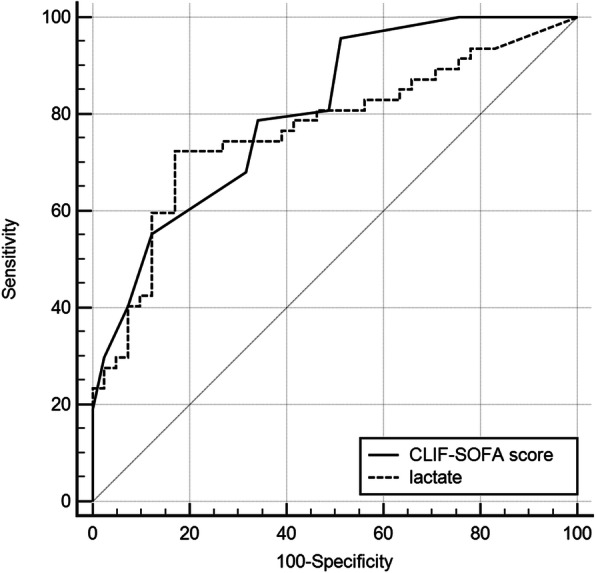
Receiver operating characteristic curves for 30-day mortality prediction of CLIF-SOFA score and lactate levels in cirrhotic patients with septic shock

We used a multivariate Cox regression model to identify independent factors associated with 30-day in-hospital mortality risk in cirrhotic patients with septic shock. As shown in Table 3, CLIF-SOFA score (adjusted hazard ratio [AHR] =1.52, *p* < 0.01), serum lactate (AHR =1.03, *p* < 0.01), and source control (AHR =0.54, *p* = 0.02) remained independent factors associated with 30-day in-hospital mortality prediction. The achievement of hour-1 bundle was not associated with mortality risk in the multivariate model (AHR =0.38, *p* = 0.08). We further compared the mortality discriminative ability between the CLIF-SOFA and lactate by using ROC curves. As shown in Fig. 3, the area under the curve (AUC) of CLIF-SOFA and lactate in mortality discrimination were 0.81 (95% confidence interval [CI] = 0.72–0.90, *p* < 0.001) and 0.77 (95% CI = 0.67–0.87, *p* < 0.001), respectively. The calculated cutoff value for the best mortality discriminative ability of CLIF-SOFA score is 11, and 4.56 mmol/L for the lactate.

**Table 3:** Multivariate Cox regression analysis of factors associated with 30-day mortality in cirrhotic patients with septic shock (n = 88)

Variables	Adjusted hazard ratio	95% CI	*p* value
Age (year)	1.03	0.97–1.11	0.13
Sex (male)	1.14	0.96–1.35	0.21
Respiratory tract infection	3.17	0.83–5.83	0.59
Biliary tract infection	0.53	0.18–1.12	0.21
CLIF-SOFA score	1.52	1.19–1.95	< 0.01^*^
Lactate	1.03	1.01–1.05	< 0.01^*^
Source Control (yes)	0.54	0.14–0.75	0.02^*^
Hour-1 bundle achievement (yes)	0.38	0.29–1.05	0.08

*P < 0.05

## Discussion

In this ED-based single-center retrospective study, we investigated the clinical characteristics of patients with cirrhosis and septic shock, and demonstrated a substantially high mortality rate of these patients regardless of whether the SSC hour-1 bundle was implemented or not. We also highlighted that the serum lactate level and CLIF-SOFA score were independent and good predictors of mortality risk in cirrhotic patients with septic shock. Furthermore, although were not included in the SSC hour-1 bundle, the measures of source control influenced the outcomes of these patients.

Although there are advances in the management of both septic shock and cirrhosis in recent years, management for patients with cirrhosis and septic shock remains challenging to clinicians; with an estimated ranging of 60 to 80% short-term mortality rate [10–12]. Patients with cirrhosis are in a state of excessive activation of pro-inflammatory cytokines (e.g., TNF-α and IL-6), decreased phagocytic activity and complement synthesis, impaired neutrophil function, and Kupffer cell production, that predisposes them to bacterial infection and sepsis, which further leads to sepsis-induced organ failure and mortality [4, 19, 20]. It becomes more complicated that patients with cirrhosis have lower baseline blood pressure and heart rate, and lack of fever or conventional inflammatory markers elevation, making identification of sepsis or septic shock in patients with cirrhosis difficult and often delayed [9, 21].

Half of our patients had cirrhosis attributed to alcohol misuse, which was different from a recent epidemiological study of chronic liver disease and cirrhosis [22]. Interestingly, alcohol was reported to be associated with altered innate and adaptive immune reactions and diminished bacterial and endotoxin clearance, resulting to the immunocompromised status of patients with alcohol use disorder, more susceptible to bacterial infection [23]. Chronic alcohol use also contributes to worsening organ failure and was identified as an independent risk factor for septic shock [24].

The distribution of infection source proportion in our cohort was similar to that in previous studies [9–12]. Unlike other subgroups that were more likely to have respiratory or urinary tract infections as sources of sepsis, patients with cirrhosis are predisposed to spontaneous bacterial peritonitis due to their intestinal bacterial overgrowth, intestinal dysmotility and increased intestinal permeability, leading to bacterial translocation, even bacteremia [20]. Moreover, the event of spontaneous bacterial peritonitis is one of the important prognostic landmarks in the natural course of liver cirrhosis, causing a high mortality rate in hospitalized patients [3, 20].

The proportion of inappropriate antimicrobial agent administration in our study was also similar to that in previous studies, with a trend of increasing nowadays [9, 10, 25]. The increasing prevalence of multi-drug resistant bacteria yield in cirrhotic patients due to the widespread antimicrobial agent exposure is another important issue needs to be addressed, since infection caused by multi-drug resistant bacteria has been prospectively validated as an independent factor associated with treatment failure and mortality [25, 26].

Our study failed to demonstrate a significant survival benefit with application of the SSC hour-1 bundle in cirrhotic patients with septic shock. Less than one-third of our patients who achieved the hour-1 bundle further highlighted the difficulty of sepsis/septic shock identification in cirrhotic patients, and implementation of *all* components of the bundle in patients within 1 h seems beyond the capacity of a crowding ED [27]. The SSC 6-h bundle did not improve the survival rate of patients with cirrhosis and septic shock in a previous prospective observational study [8]. It was proposed that the hypoalbuminemia and vasodilated state of cirrhotic patients made the evaluation of fluid status and resuscitation response more difficult [8]. Compared with the initial 30 ml/kg crystalloid administration suggested by SSC, the relatively lower amount of intravenous fluid resuscitated in our patients was in consideration to their risk of interstitial and pulmonary edema [8]. Besides, the relatively lower baseline blood pressure in cirrhotic patients made the decision of early support of vasopressors for their hypotension more challenging [4]. In addition, the survival benefit of early administration of antimicrobial agents in septic shock patients has been questioned in several meta-analysis studies [28, 29]. Taken together, little evidence exists to support that each component of the hour-1 bundle is equally effective and can be applied with equal consistency, especially in a physiologically unique and fragile cirrhosis group [27, 30].

Compared with other measures in treating cirrhotic patients with sepsis and septic shock, the literature seldom emphasizes the importance of source control [4, 18]. A prospective observational study revealed that a lower mortality risk was observed in septic patients who underwent source control regardless of the timing of intervention [31]. Our patients in the survivor group, who had a significantly higher proportion of biliary tract infection, echoed this finding since they had a higher chance of receiving measures of source control (e.g., percutaneous, endoscopic, or surgical intervention). The independent decreased mortality association of source control implementation in our study also reminds clinicians the prognostic significance of this maneuver while treating cirrhotic patients with septic shock.

Our study identified serum lactate levels and CLIF-SOFA scores as independent mortality risk predictors in cirrhotic patients with septic shock. Lactate has been recognized as a marker of tissue hypoxia and systemic hypoperfusion, and has been validated as a prognostic factor associated with organ failure and short-term mortality in critically ill patients with liver cirrhosis in a recent large cohort study [32]. Similarly, the CLIF-SOFA score, which is modified for patients with end-stage liver disease prognosis stratification [15], was identified as an independent short-term outcome predictor for cirrhotic patients in ICU [33]. The score of 11 in the previous study as the mortality discriminative value was also consistent with our findings [33]. Our findings need further larger prospective studies to validate their prognostic values in these patients.

Our study had several limitations. First, the retrospective nature of this study made the recall and selection bias inevitable. Second, this was conducted in a single center, which may limit the generalization of these results to other institutions. Third, we only enrolled cirrhotic patients with septic shock and not those with less severe (i.e., sepsis without shock) diagnosis. This could misinterpret our validation of hour-1 bundle results since they were proposed for all patients with sepsis and septic shock [6].

## Conclusions

In conclusion, cirrhotic patients with septic shock have a substantially high mortality rate, and the application of the hour-1 bundle proposed by SSC did not reveal a significant survival benefit to them. The serum lactate level, CLIF-SOFA score, and source control implementation were independent factors associated with prognosis of these patients. Clinicians could put more emphasis on them in patients with cirrhosis and septic shock.

## Data Availability

The datasets used and/or analyzed during the present study are available from the corresponding author upon reasonable request.

## Abbreviations

ACLF: Acute-on-chronic liver failure
SSC: Surviving sepsis campaign
ED: Emergency department
MAP: Mean arterial pressure
MELD: Model for End-stage Liver Disease
CLIF-SOFA: Chronic liver failure-sequential organ failure assessment
SIRS: Systemic Inflammatory Response Syndrome
qSOFA: Quick Sepsis Related Organ Failure Assessment
ROC: Receiver operating characteristic
INR: International normalized ratio
